# Effects of surgery on free and total 25 hydroxyvitamin D concentrations in dogs

**DOI:** 10.1111/jvim.15933

**Published:** 2020-10-14

**Authors:** Dylan N. Clements, Gemma Bruce, John M. Ryan, Ian G. Handel, Ioannis L. Oikonomidis, Adam G. Gow, Helen Evans, Susan Campbell, Emma Hurst, Richard J. Mellanby

**Affiliations:** ^1^ Royal (Dick) School of Veterinary Studies and The Roslin Institute The University of Edinburgh, Hospital for Small Animals Roslin, Midlothian UK; ^2^ Nationwide Specialist Laboratories Unit 2 Sawston Park Cambridge, Cambridgeshire UK

**Keywords:** 25 hydroxyvitamin D, cruciate rupture, dog, inflammation, vitamin D

## Abstract

**Background:**

It is unclear whether a low total 25(OH)D concentration is a cause or consequence of illnesses. To address this knowledge gap, studies measuring free and total 25(OH)D during the evolution and resolution of an inflammatory process are required.

**Objectives:**

Serum total and free 25(OH)D concentrations would transiently decline after cruciate surgery in dogs.

**Animals:**

Seventeen client‐owned dogs with a spontaneous cranial cruciate ligament rupture (CCLR).

**Methods:**

A longitudinal cohort study involving the measurement of serum concentrations of total and free 25(OH)D, total calcium, creatinine, albumin, phosphate, C‐reactive protein and plasma ionized calcium, at 1 day before and a median time of 1 and 60 days after surgical treatment of CCLR.

**Results:**

Median serum concentrations of total 25(OH)D before surgery (80.3 nmoL/L [range, 43.5‐137.3]) significantly declined immediately after surgery; (64.8 nmoL/L [range, 36.3‐116.5] 1 day after surgery, *P* < .005) before increasing to become nonsignificantly different from concentrations before surgery at day 60 after surgery (median 78.0 nmoL/L [range, 24.2‐115.8], *P* = .14). In contrast, median free 25(OH)D concentrations before surgery (7.6 pg/mL [range, 3.8‐12.2]) significantly increased immediately after surgery (9.2 pg/mL [range, 5.2‐15.7], *P* < .05) before declining to become nonsignificantly different from before surgery concentrations at day 60 after surgery (median 6.2 pg/mL [range, 4.0‐15.8], *P* = .37).

**Conclusion and Clinical Importance:**

This study reveals the difficulties of assessing vitamin D status in dogs following elective surgery.

**Abbreviations**
25(OH)D: 25 hydroxyvitamin D
CCLR: cranial cruciate ligament rupture
CCWO: cranial closing wedge osteotomy
CRP: C‐reactive protein
PTH: parathyroid hormone
TPLO: tibial plateau leveling osteotomy
VDR: vitamin D receptor

## 1 INTRODUCTION

Vitamin D has a well‐established role in the development and maintenance of skeletal health.^1^, ^2^Over the past decade, interest in the nonskeletal effects of vitamin D in dogs has increased, partly due to a growing recognition that many nonskeletal tissues express the vitamin D receptor (VDR).^3^ Dogs with systemic illnesses have low serum total 25 hydroxyvitamin D (25[OH]D) concentrations.^4^, ^5^, ^6^, ^7^, ^8^, ^9^, ^10^ It remains unclear whether a low total 25(OH)D concentration predisposes to the development of nonskeletal disorders or is actually a consequence of illness. There is a negative correlation between total 25(OH)D concentrations and inflammatory biomarkers in dogs with nonskeletal diseases.^11^, ^12^ Similarly in healthy dogs, there is a negative relationship between serum total 25(OH)D concentrations and inflammation.^13^

However, it is unknown whether low serum 25(OH)D concentrations in dogs predispose to the development of inflammation or is simply a secondary consequence of an inflammatory state. A small number of longitudinal studies have been performed, which have serially measured total 25(OH)D concentrations in dogs during the development and resolution of systemic inflammation.^14^, ^15^ These studies have yielded conflicting results and there is an absence of studies that have examined both free and total 25(OH)D concentrations, alongside other key calcium homeostasis metabolites, during the development of an inflammatory response in dogs. Dogs undergoing surgical treatment of a spontaneous cranial cruciate ligament rupture (CCLR) are an ideal population in which to longitudinally track total and free 25(OH)D concentrations during an inflammatory process since dogs with a CCLR are typically clinically stable before surgery and frequently develop systemic inflammation shortly after surgery, which subsides in dogs that recover from the procedure.^16^ Therefore, the objective of this study was to longitudinally track total and free 25(OH)D and C‐reactive protein (CRP) concentrations, together with ionized calcium, total calcium, phosphate, albumin, and creatinine concentrations, in clinically stable dogs undergoing elective surgical treatment of their CCLR.

## 2 METHODS

All dogs diagnosed with a CCLR at the Hospital for Small Animals, University of Edinburgh, between June 2015 and December 2017 were considered eligible for inclusion in the study. Entry criteria included a confirmed diagnosis of CCLR and owner consent to take a blood sample the day before surgery, 1 day after the surgical treatment of the CCLR, and at the 8 week after surgery re‐examination. The surgical treatment method undertaken was decided upon by the attending clinician and was either a tibial plateau leveling osteotomy (TPLO) or cranial closing wedge osteotomy (CCWO).

Blood samples collected at each timepoint were placed into plain, ethylenediaminetetraacetic acid (EDTA), and lithium heparin blood collection tubes, which were refrigerated after collection. Albumin, total calcium, ionized calcium, creatinine, and inorganic phosphate were all measured within 3 hours of collection. Serum and EDTA plasma were separated by centrifugation within 3 hours of collection and aliquoted for subsequent batch analysis of total and free 25(OH)D and parathyroid hormone (PTH), respectively, and stored frozen at −80°C until analysis.

Albumin, total calcium, creatinine, inorganic phosphate, and CRP concentrations were measured using an Au480 Chemistry Analyser (Beckman Coulter [UK] Ltd, High Wycombe, UK). The following assays were used for the measurement of the abovementioned analytes: bromocresol green for albumin, Arzenazo for total calcium, modified Jaffe for creatinine, phosphomolybdate complex for inorganic phosphate, and a species‐specific immunoturbidimetric assay for CRP. Ionized calcium was analyzed on the GEM Premier 3500 (Instrumentation Laboratory, Massachusetts) using ion‐selective electrode potentiometry. Plasma PTH concentrations were measured using a previously validated 2‐site immunoradiometric assay by NationWide Specialist Laboratories (Cambridge, UK).

Serum concentrations of total 25(OH)D_2_ and 25(OH)D_3_ were measured by liquid chromatography tandem mass spectrometry (LC‐MS/MS) as previously described by the Vitamin D Animal Laboratory (VitDAL), which has been certified as proficient by the international Vitamin D Quality Assurance Scheme (DEQAS).^17^ The results are presented as total 25(OH)D, which is defined as the sum of 25(OH)D_2_ and 25(OH)D_3_.

Free 25(OH)D was measured using the Free 25OH Vitamin D ELISA (DIAsource ImmunoAssays S.A, Belgium) as previously described.^18^ This 2‐step quantitative immunoassay was carried out following manufacturer's instructions using 10 μL of serum. Absorbance was measured at 450 nm using a plate spectrophotometer (WPA Biowave, Biochrom) with Viktor3 software (PerkinElmer). The concentration of free 25(OH)D in the sample was inversely proportional to absorbance.

For each metabolite, the 2 after surgery blood sample results were compared to the before surgery blood sample by a Wilcoxon matched pairs signed‐rank test. Significance was set at *P* < .05.

The study was approved by the University of Edinburgh Animal Welfare and Ethics Review Board.

## 3 RESULTS

Seventeen dogs were enrolled in the study. The median age of the dogs was 6.7 years and ranged from 2.1 years to 11.6 years. There were 8 neutered females, 8 neutered males, and 1 entire female. The cohort consisted of 6 Labradors, 5 Crossbreed dogs, 2 Staffordshire Bull Terriers, 1 Border Terrier, 1 Bulldog, 1 Cocker Spaniel, and 1 Dogue de Bordeaux. The median weight of the dogs was 26.5 kg (range, 16.5‐38.8 kg). No dogs were known to have been administered calcium or vitamin D supplements prior to enrolment onto the study. Ten dogs had a rupture of the right and 7 dogs had a rupture of the left cranial cruciate ligament. A TPLO surgical procedure was undertaken in 11 dogs and a CCWO was performed in 6 dogs. No cases experienced a significant surgical hemorrhage during the procedure, and the volume of blood lost in each case was estimated to be <5%. Dogs were administered fluids intravenously (10 mL/kg/h) intraoperatively and for 2 to 3 hours after surgery.

Blood samples were collected 1 day before surgery and a median time of 1 day and 60 days after surgery. Ten dogs were blood sampled at day 1 after surgery with the 7 other dogs sampled at day 2 after surgery. At the second follow‐up timepoint, 16 of the 17 dogs were re‐examined between 48 and 69 days after surgery. The final case was examined 140 days after surgery. Serum calcium, albumin, phosphate, creatinine, 25(OH)D, and ionized calcium concentrations were available from all 17 dogs. Plasma PTH measurements were available in 16 dogs, and in 1 case there was insufficient plasma to run the assay. The serum concentrations of CRP were within the reference interval in 16 of the 17 dogs before surgery before significantly increasing in all dogs at the 1 day after surgery timepoint. At the 60 day after surgery re‐evaluation, the CRP concentrations had returned to within the reference limit in almost all dogs (Table 1).

**TABLE 1:** Median, lower, and upper values of serum C‐reactive protein, total calcium, phosphate, albumin, creatinine, total 25(OH)D, free 25(OH)D, and plasma ionized calcium and parathyroid hormone at day −1, day 1, and day 60 after surgery in 17 dogs presented for cranial cruciate ligament rupture

Analyte (reference interval)	Day −1	Day 1 after surgery	Day 60 after surgery
C reactive protein mg/L (0‐5.0)	5.0 (5.0‐10.2)	50.3 (13.3‐79.7)^a^	5.0 (5.0‐6.1)
Total calcium mmol/L (2.24‐2.85)	2.62 (2.37‐2.85)	2.47 (1.61‐2.63)^b^	2.58 (2.44‐2.79)
Ionized calcium mmol/L (1.18‐1.53)	1.34 (1.22‐1.54)	1.38 (1.26‐1.51)	1.36 (1.25‐1.55)
Phosphate mmol/L (0.90‐2.00)	1.05 (0.55‐2.08)	1.43 (1.23‐1.81)^c^	1.02 (0.61‐1.45)
Albumin g/L (27.0‐38.8)	34.1 (30.2‐39.5)	32.8 (29.1‐35.9)^b^	34.7 (26.9‐39.4)
Creatinine μmol/L (22.0‐115.0)	113.0 (69.0‐129.0)	88.0 (65.0‐115.0)^a^	119.0 (81.0‐142.0)
Parathyroid hormone pg/mL (20.0‐65.0)	45.5 (10.0‐165.0)	63.5 (10.0‐175.0)	62.0 (14.8‐110.0)
Total 25(OH)D nmol/L (35.1‐152.2)	80.3 (43.5‐137.3)	64.8 (36.3‐116.5)^d^	78.0 (24.2‐115.8)
Free 25(OH)D pg/mL (2.4‐14.2)	7.6 (3.8‐12.2)	9.2 (5.2‐15.7)^c^	6.2 (4.0‐15.8)

Abbreviation: 25{OH)D, 25 hydroxyvitamin D.

^a^ Significant difference between day −1 value *P* < .0001.

^b^ Significant difference between day −1 value *P* < .0005.

^c^ Significant difference between day −1 value *P* < .05.

^d^ Significant difference between day −1 value *P* < .005.

The concentrations of plasma ionized calcium and PTH and serum total calcium, phosphate, albumin, and creatinine at the 3 timepoints are shown in Table 1. The plasma ionized calcium and PTH concentrations did not significantly differ from their respective baseline concentrations at either timepoint after surgery. In contrast, the albumin, creatinine, and total calcium concentrations significantly decreased, albeit modestly, following surgery before returning to near baseline values 60 days after surgery (Table 1). Phosphate concentrations increased immediately after surgery before declining to become nonsignificantly different from baseline values 60 days after surgery (Table 1).

Serum total 25(OH)D concentrations significantly declined 1 day after surgery, but the 60 days after surgery concentrations were not statistically different from baseline (Table 1). In contrast, serum free 25(OH)D significantly increased 1 day after surgery before declining to nonstatistically different concentrations at 60 day after surgery re‐evaluation (Table 1). At day 1 after surgery re‐evaluation, serum total 25(OH)D concentrations had declined in 13 of the 17 dogs, whereas serum‐free 25(OH)D concentrations had increased in 12 of the 17 dogs. None of the total 25(OH)D concentrations were outside the reference interval the 1 day after surgery and only 2 dogs had a free 25(OH)D concentrations outside the reference interval 1 day after surgery; both values were slightly above the upper reference limit.^18^

## 4 DISCUSSION

The central finding of this study was that dogs undergoing elective orthopedic surgery, which caused an acute inflammatory response as characterized by a sharp increase in CRP, experienced a transient decline in total 25(OH)D concentrations and an increase in serum‐free 25(OH)D concentrations. The decline in serum total 25(OH)D concentrations after an inflammatory response is consistent with experimental studies in dogs where 25(OH)D and ionized calcium concentrations declined and PTH concentrations increased following the intravenous administration of lipopolysaccharide.^15^ However, in our study, we found no significant changes in ionized calcium or PTH concentrations at either postoperative time periods, suggesting that the decline in total 25(OH)D was not sufficient to cause clinically relevant changes in calcium homeostasis in dogs. In addition, the decline in total 25(OH)D concentrations and increase in free 25(OH)D concentrations were relatively modest with no total 25(OH)D values 1 day after surgery falling below the lower reference intervals.^18^

Similar to research findings in dogs,^11^, ^12^, ^13^ there is a negative relationship between biomarkers of inflammation and serum total 25(OH)D concentrations in people.^19^, ^20^, ^21^, ^22^ This has resulted in several studies examining vitamin D status during the initiation and resolution of an inflammatory response in human patients. A systematic review of longitudinal studies of serum total 25(OH)D concentrations during an acute inflammatory response identified 8 studies, which met the entry criteria of the review.^23^ In 6 of these studies, total 25(OH)D concentrations declined after the inflammatory insult while in the other 2 studies there was no change in total 25(OH)D during the course of the disease. However, in the latter 2 studies, the interpretation was limited as baseline total 25(OH)D concentrations were measured after onset of symptoms.^23^ Similar to the findings reported in this study, the 3 studies that longitudinally tracked vitamin D status in humans undergoing orthopedic surgery found that 25(OH)D concentrations declined in the immediate postoperative period alongside an increase in CRP concentrations.^24^, ^25^, ^26^ Again, similar to our findings, PTH concentrations did not significantly change after knee arthroplasty supporting the concept that the decline in 25(OH)D concentrations do not cause clinically relevant disturbances in calcium homeostasis.^25^

The cause of the transient postoperative decline in serum total 25(OH)D concentrations in our study is unclear. Serum vitamin D binding protein decreased after elective orthopedic surgery.^24^, ^25^ Consequently, a decline in vitamin D binding protein in dogs after surgery could potentially explain the decline in total 25(OH)D concentrations observed in this study. Another potential explanation of the observed transient decline in total 25(OH)D concentrations after surgery may be due to hemodilution because of fluid therapy, which was administered before, during, and after surgery. The concurrent decline in postoperative creatinine concentrations suggests that hemodilution after fluid therapy could be at least partly responsible for the decline in albumin and 25(OH)D concentrations. A decline in 25(OH)D concentrations after acute fluid shifts occurs in human ICU patients.^27^ However, this study longitudinally tracked 25(OH)D concentrations following significant hemodilution during cardiopulmonary bypass procedures and so did not mimic the clinical situation of isotonic fluid therapy associated with elective surgery in our study. The decline in total, but not ionized, calcium could reflect the decline in albumin concentration, which reduced the protein‐bound calcium component.^28^, ^29^

Although the relationship between 25(OH)D and nonskeletal diseases has been examined extensively, there are limited data on the relationship between directly measured free 25(OH)D concentrations, inflammation, and nonskeletal health in either human or veterinary patients. In dogs, less than 1% of total serum 25(OH)D is available as free 25(OH)D.^18^

A small number of cross‐sectional studies in humans have reported serum total 25(OH)D and directly measured free 25(OH)D concentrations in several patient groups which has revealed that patients with low total 25(OH)D concentrations often have free 25(OH)D within or above the reference interval. Free 25(OH)D is increased in human liver patients despite lower 25(OH)D concentrations compared to healthy controls.^30^ Similarly an increase in free 25(OH)D alongside a decrease in serum 25(OH)D concentrations occurs in pediatric patients with inflammatory bowel disease.^31^ In our study, we speculate that the increase in free 25(OH)D concentrations could explain why ionized calcium and PTH concentrations were not significantly different from baseline after surgery despite the decline in total 25(OH)D concentrations. As the concentration of total 25(OH)D decreases, the relative percentage of free 25(OH)D could be increased in order to maintain homeostasis of 1,25 dihydroxyvitamin D, ionized calcium, and PTH. Limitations of our study include the relatively small number of dogs enrolled, only using 3 time points to assess vitamin D metabolite concentrations and not assaying a more comprehensive panel of vitamin D metabolites. Another limitation was that although no dogs had evidence of systemic disease other than a CCLR based on history and physical examination, the lack of extensive before surgery diagnostic investigations means that the presence of other concurrent, subclinical diseases cannot be definitively excluded.

## CONFLICT OF INTEREST DECLARATION

Authors declare no conflict of interest.

## OFF‐LABEL ANTIMICROBIAL DECLARATION

Authors declare no off‐label use of antimicrobials.

## INSTITUTIONAL ANIMAL CARE AND USE COMMITTEE (IACUC) OR OTHER APPROVAL DECLARATION

Approved by the University of Edinburgh Animal Welfare and Ethics Review Board (project license number 7007937).

## HUMAN ETHICS APPROVAL DECLARATION

Authors declare human ethics approval was not needed for this study.
